# Concrete Cover Cracking and Reinforcement Corrosion Behavior in Concrete with New-to-Old Concrete Interfaces

**DOI:** 10.3390/ma16175969

**Published:** 2023-08-31

**Authors:** Juhui Zhang, Jing Li, Yuchuan Zhao, Shikun Wang, Zhongguo Guan

**Affiliations:** 1 Department of Civil Engineering, University of Shanghai for Science and Technology, Shanghai 200093, China; jing_ls@126.com; 2 Tongji Architectural Design (Group) Co., Ltd., Shanghai 200092, China; zhaoyuchuan@tjad.cn; 3 Shanghai Construction Engineering Group Co., Ltd., Shanghai 200080, China; 19821231708@163.com; 4 State Key Laboratory for Disaster Reduction in Civil Engineering, Tongji University, Shanghai 200092, China; guanzhongguo@tongji.edu.cn

**Keywords:** new-to-old concrete interface, corrosion of reinforcement, concrete surface cracking, sectional loss, RC structures

## Abstract

In reinforced concrete (RC) structures, new-to-old concrete interfaces are widely present due to precast splices, repairs, and construction joints. In this paper, both monolithic and segmental specimens were fabricated with five kinds of water–cement ratios, including ordinary and high-strength concrete. The impressed current-accelerated corrosion test was used, and the degree of reinforcement corrosion was controlled by Faraday’s Law. In the accelerated corrosion process, the concrete surface cracking, steel corrosion, and mechanical properties of the corroded steels in the segmental specimens were investigated and compared with monolithic specimens considering the pouring method, concrete strength, and the strength difference between new and old concrete. The prediction of concrete cracking time was also discussed. The results indicated that, for the monolithic specimens, longitudinal cracks could be observed on the ordinary concrete surface, while no cracks were produced on a high-strength concrete surface; only the rust leaked out at the ends. For the segmental specimens, both longitudinal and transverse cracks were produced on an ordinary concrete surface, while only transverse cracks were produced at the high-strength new-to-old concrete interfaces. The steel embedded in the segmental specimens suffered more sectional loss at the new-to-old concrete interfaces. An influence coefficient based on the section loss of the rebar was proposed to evaluate the influence of interfaces on the rust uniformity of rebars. When there were differences in strength between new and old concrete, the influence of the interface on the uniformity of steel bar cross-section loss slightly increased. Based on available theoretical analysis for uniform corrosion, the concrete cracking time of the monolithic specimens was predicted, which was basically consistent with experimental phenomena. However, further research is needed to predict the service life of segmental specimens with new-to-old concrete interfaces.

## 1. Introduction

Steel corrosion in concrete has become the most significant cause of structural deterioration, especially for marine structures. Chloride ions in the marine environment can penetrate the concrete cover and make the steel depassivate and lose the protective oxide film. Once corrosion initiates, corrosion products will continuously accumulate around the reinforcement, leading to the cracking and spalling of the surrounding concrete and causing the degradation of RC structures [1,2,3]. Therefore, it is important to consider steel corrosion and its effects on concrete surface cracking and propagation. Many studies have been carried out to investigate the effect of reinforcement corrosion on concrete cracking. Andrade et al. [4] and Alonso et al. [5] investigated the loss of bar cross-section needed to induce concrete cover cracking and developed a numerical model to relate the decrease in the rebar cross-section to the cover cracking. Vidal et al. [6] and Zhang et al. [7] put forward theoretical models to predict steel corrosion from corrosion cracking using the parameter of steel cross-sectional loss based on experimental results over ten years. Malumbela et al. [8,9] conducted experimental studies to investigate the relationship between the rate of crack widening and the crack pattern, as well as the level of steel corrosion for corroded RC beams under various levels of sustained loads. Zhao et al. [10] investigated a reinforced concrete specimen that had deteriorated in an artificial environment for 2 years to investigate the millscale on the rebar surface and the distribution of rust using SEM to observe the steel/concrete interface and corrosion-induced cracks. Rivera-Corral et al. [11] exposed specimens to the penetration of chlorides using the dry–wet cycling method for 2.6 years. The corrosion behavior of steel bars with different surface finishes (ordinary (CS), dual phase (TTS), and galvanized (GS)) was studied using prismatic concrete specimens with two water/cement (w/c) ratios: 0.45 and 0.65. Wu et al. [12] used electrochemical techniques and scanning electron microscopy to study different steel bars in a simulated concrete pore solution over different periods of time. Cai et al. [13] designed reinforced concrete specimens with vertically and horizontally arranged steel bars of different heights and exposed them to wetting/drying cycles in chloride solution by using the open circuit potential (OCP) of the steel bars to monitor the corrosion state. Wang et al. [14] found that the addition of fly ash microspheres (FAMs) to concrete improved the interfacial transition zone and blocked the connected porosity of the hardened cement paste. The filler effect of the FAM particles significantly improved the pore structure, which increased the compressive strength and chloride penetration of the concrete. Oliveira et al. [15] studied the effect of coarse aggregate size on the degree of corrosion of reinforcement in concrete by the electrochemical techniques under the coupling of two factors, carbonation and chloride ion attack. Concrete with 25 mm coarse aggregate exhibited the worst results in the electrochemical tests, indicating a concrete/reinforcement interface incapable of retaining electrical charges, with low resistance to corrosion progress. Zhang et al. [16] found that the addition of monosulfate hydrate (AFm) increased the chloride threshold value (CTV) for steel corrosion. After reaching the CTV, the corrosion rate of steels increased dramatically due to the release of the bound Cl^−^. Zhang et al. [17] studied the rust expansion cracking behavior of Cr alloy-reinforced coral aggregate concrete (CAC) using theoretical and experimental methods to analyze the serviceability limit state of concrete. The corrosion product composition of the Cr alloy rebar was analyzed to determine the stability and protection of the rust layer. The above research shows that there are many factors that influence concrete cracking due to reinforcement corrosion, which may exist simultaneously and influence each other: (1) environmental factors, such as temperature, humidity, and aggressive media; (2) rust expansion, which is more likely to crack in weak areas of concrete; (3) the characteristics of the reinforcement–concrete interface, (4) external loads, etc. However, the above studies mainly focused on monolithic reinforced concrete structures.

In practical engineering, new-to-old concrete structures are widely present due to precast splices, repairs, and construction joints. If reinforcement corrosion occurs at the new-to-old concrete interface, it inevitably leads to a serious impact on the bond strength of the interface and further endangers structural safety. Zhao et al. [18] found that the chloride content in the interfacial zone is higher than that elsewhere, which shows the interfacial zone effect (IZE). The interfacial zone of new–old concrete is the natural weak part in durability, and fatigue stress and an aggressive medium will accelerate the durability degradation of the interfacial zone. Zhang et al. [19] demonstrated experimentally that aggressive ions tended to penetrate the concrete through the interfacial transition zone between the joint interfaces and easily cause the corrosion of inner rebars. Studies have shown that chloride erosion is more severe at concrete joints, which deserves attention. Li et al. [20] studied the effect of joint type and load conditions on the resistance of segmental joints to chloride ions through salt solution immersion tests. Liu et al. [21] investigated the effect of reinforcement corrosion on the shear strength of the new-to-old concrete interface based on accelerated corrosion tests. Yoo and Yang et al. [22,23] investigated the effect of load level and slag powder on chloride ion transport in cold joints using the electrical acceleration method and found that the chloride ion transport rate was significantly accelerated at the joints. Li et al. [24] performed chloride ion intrusion on four types of joints commonly used in segmentally formed bridge concrete structures, including direct wet joints, roughened wet joints, dry joints, and epoxied joints. Although the durability performance of the different types of joints varied, the degree of corrosion was greater than that of the non-jointed structures. Huang et al. [25] conducted a corrosion test on 90 specimens by the electrochemical accelerated corrosion method to assess the effect of construction joints on the chloride-induced corrosion of reinforcing steel in concrete. The test parameters included two environmental conditions (salt solution immersion condition and cyclic wet–dry condition), two forms of construction joint (direct wet joint and roughened wet joint), and four types of steel bar (mild steel bar, ferritic stainless-steel bar, austenitic–ferritic stainless-steel bar, and epoxy-coated steel bar). In summary, the mechanism of chloride ion transport between new and old concrete is complex. The corrosion rate affecting the new-to-old concrete interface is relatively similar to that of monolithic concrete and is influenced by several interacting parameters: the water–cement ratio, the type of reinforcement, the loading, etc. Also, the properties of the new-to-old concrete interface affect the corrosion rate, such as the roughness of the interface and the form of interfacial bonding.

However, existing research has rarely considered the surface crack propagation and steel corrosion behavior caused by the presence of new-to-old concrete interfaces. Thus, in this study, the differences in surface cracking and reinforcement corrosion between monolithic and segmental specimens were comprehensively compared, revealing the morphology and generation mechanism of concrete surface cracking. The effect of concrete strength on the durability of RC structures with new-to-old concrete interfaces was also investigated. Additionally, the prediction of the concrete cracking time was also discussed to provide practical recommendations for engineering practice.

## 2. Experimental Program

### 2.1. Preparation of Specimens and Mixed Proportions

**Figure 1:**
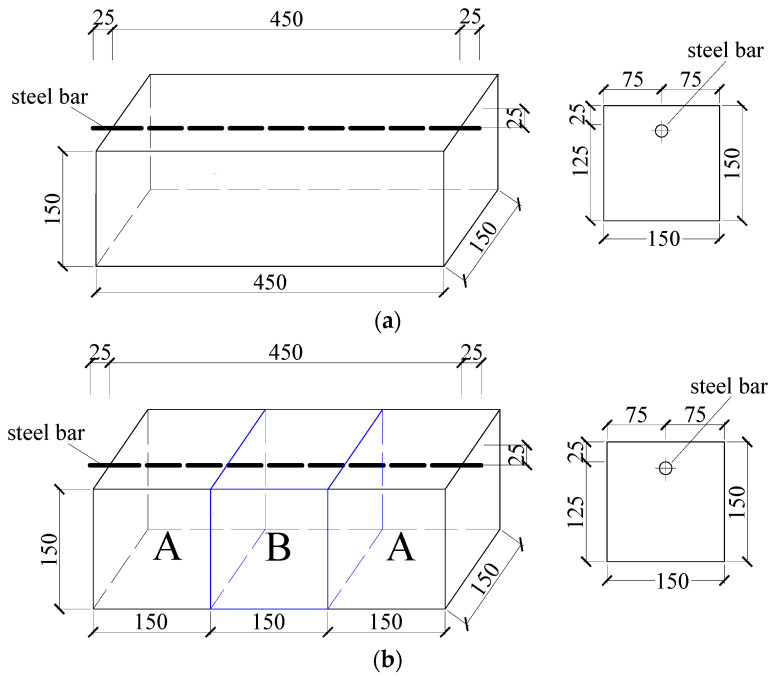
Specimen design: (**a**) Monolithic specimens; (**b**) Segmental specimens (Unit: mm).

**Figure 2:**
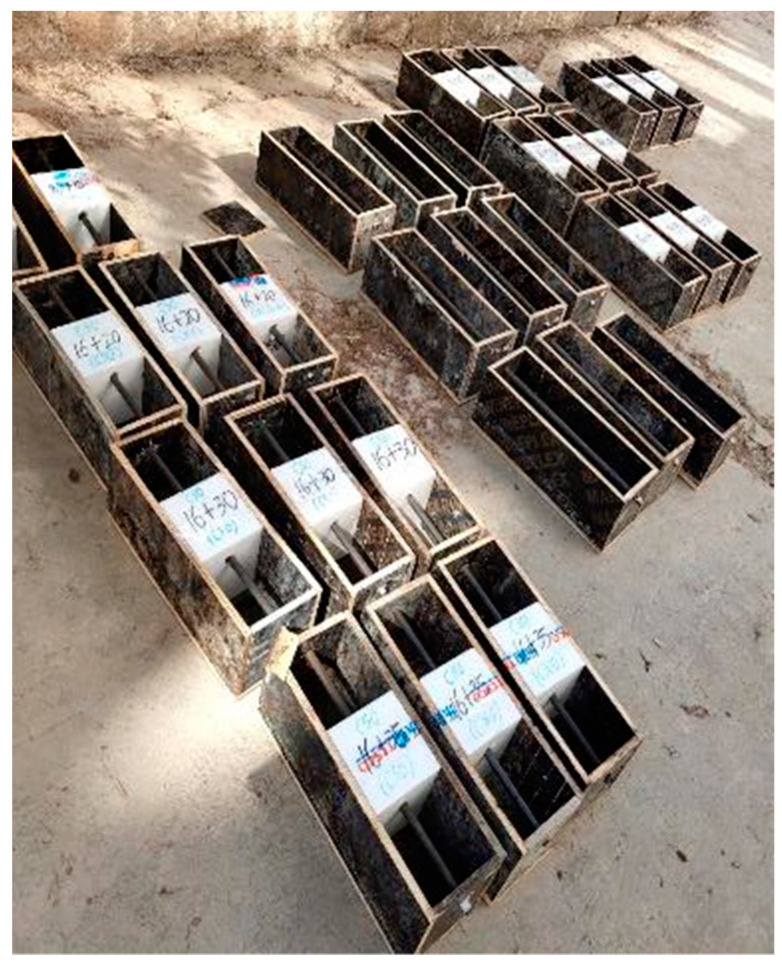
Steel bars embedded in the molds.

In this study, both monolithic and segmental specimens with new-to-old concrete interfaces were fabricated, as shown in Figure 1. The size of all specimens was 150 × 150 × 450 mm and each specimen was reinforced with one 16 mm steel bar with a net cover depth of 25 mm. For the segmental specimens, as shown in Figure 2, in addition to a prefabricated wooden mold with a size of 150 × 150 × 450 mm, a cubic foam mold with a size of 150 × 150 × 150 mm was also prepared. Holes were first drilled on both the foam and wooden molds according to the rebar diameter and concrete cover thickness, and then the foam mold was placed at the location of part B. A 500 mm long steel bar, which had been manually de-rusted and weighed, was then passed through the channel with a length of 25 mm exposed on both sides of the wooden formwork. The wires were welded at the end of the steel bar and epoxy resin was also applied to a range of 25 mm at both ends of the steel bar to seal them from corrosion. Two parts, which were denoted as part A, as shown in Figure 1b, corresponding to the old concrete, were cast in the first stage. Ten days later, the foam mold in part B was taken out, and the new-to-old concrete surface was manually roughened. After the roughening was completed, the remaining part of the specimen (i.e., part B), corresponding to the new concrete, was cast, and then both the new and old concrete was cured for 28 days.

After curing, a ZBL-F800 crack comprehensive tester was introduced to observe whether there were obvious cracks at the adhesive interface for segmental specimens. Any specimens with initially visible interface cracks were discarded and re-prepared to prevent any impact on the test results.

The influence of the casting method, concrete strength, and strength difference between new and old concrete on the corrosion of steel bars was considered in the specimen design, as shown in Table 1. Five kinds of concrete with water–cement ratios of 0.60, 0.53, 0.44, 0.33, and 0.24 were adopted in the tests, including ordinary and high-strength concrete. Cubes with dimensions of 150 × 150 × 150 mm were used for compressive strength testing. According to GB/T 50081-2019 [26], the compressive strength test was carried out on plain concrete specimens with different ratios. Three specimens of each different group were cast and placed in a standard curing box (temperature 20 ± 2 °C, relative humidity 95%) for 28 days for testing. Table 2 lists the concrete mix designs and 28-day compressive strength. Grade P.O 42.5 ordinary Portland cement was employed in concrete types No. 1, 2, and 3, while Grade P.II52.5 Portland cement was used for concrete types No. 4 and 5. The coarse aggregates were crushed stones whose particle size was between 5 mm and 20 mm, while the fine aggregates were sand. Polycarboxylate superplasticizer DSZ-1 was used as a high-range water-reducing (HRWR) admixture. Another two admixtures, Class F Grade I fly ash and S95 ground-granulated blast-furnace slag, were also added to the concrete, except for concrete type No. 1. (Class F Grade I fly ash: density ≤ 2.6 g/cm^3^, water demand ratio ≤ 95%; S95 ground-granulated blast-furnace slag: specific surface area ≥ 400 m^2^/kg, mobility ratio ≥ 95%, 28d activity index ≥ 95%).

The casting methods included integral and segmental casting. As shown in Table 1, Specimens 1-b, 5-b, 4-a, 5-a, and 6-a were monolithic specimens. The reinforcement in all specimens was HPB300 (corresponds to hot-rolled smooth-round bars with characteristic steel yield strength *f*_y_ of 300 MPa), with a uniform diameter of 16 mm and a net concrete cover thickness of 25 mm. Two bars (HPB300) were randomly selected and tested for tensile strength (averaged) with a mechanical universal machine in accordance with the requirements of specification GB/T 228.1-2021 [27]. It was used to compare with the mechanical characteristic value of the rebar after rusting. The measured yield and ultimate stresses of the uncorroded steel bar were 314 and 465 MPa, respectively.

### 2.2. Accelerated Corrosion Tests

**Figure 3:**
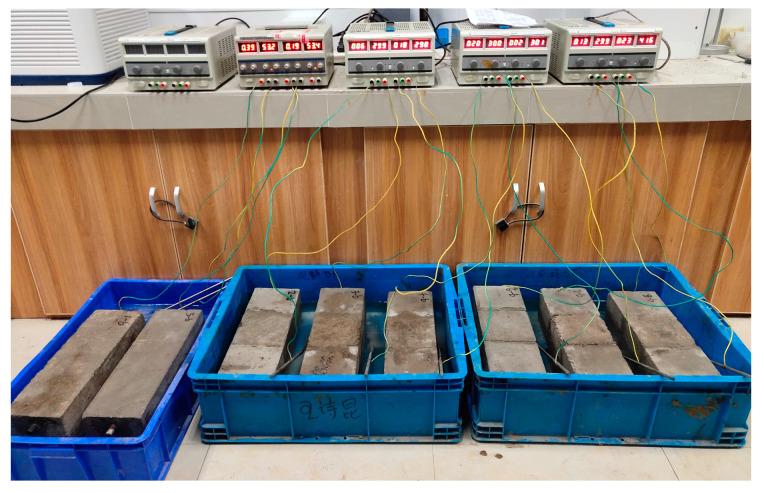
Partial immersion impressed current-accelerated corrosion.

Since the accelerated corrosion test could shorten the time significantly and the process of steel corrosion in both the accelerated corrosion and normal corrosion tests was similar [28], the impressed current-accelerated corrosion (ICAC) test was adopted for steel corrosion in the concrete specimens. Moreover, compared with the full-immersion ICAC method, since, in the semi-immersion ICAC method, one side of the concrete cover could be exposed to the air, it would be much easier to observe the crack growth and corrosion products accumulated on a concrete surface; thus, the semi-immersion ICAC method was adopted in this study. The specific procedure was as follows: (1) Specimens were first completely submerged in a 5% NaCl electrolyte solution by weight of water before the beginning of corrosion tests; (2) two days later, specimens were taken out and the surface solution was wiped off; (3) specimens were then partially submerged in the 5% NaCl solution, and the solution surface was controlled to be located below the being corroded bar embedded in the specimen, that is, the height difference between the solution surface and the bottom of the rebar was a constant value of 20 mm; (4) the steel in the specimen to be corroded was linked to the positive terminal of the direct-current (DC) power supply through welded wires, and a stainless steel rod with a diameter of 8 mm and a length of 500 mm was connected to the negative terminal. The stainless steel rod was also immersed in the 5% NaCl solution and acted as the cathode material. The corrosion current density in this study was taken as 2.5 mA/cm^2^, according to the research of Gan et al. [29], and the semi-immersion ICAC test began. A photo of the ICAC test is presented in Figure 3.

Faraday’s law was used to obtain the designed amount of reinforcement corrosion, in terms of mass loss of reinforcement:

(1) $$\Delta m_{L}=\frac{M\cdot I\cdot t}{Z\cdot F}$$

where Δ*m*_L_ is the mass loss of iron (g); *M* is the molar mass of iron (56 g/mol for Fe); *Z* is the valency of the iron element (i.e., 2.5); *F* is the Faraday constant (i.e., 96,500 C/mol); *t* is the duration of electrochemical corrosion (s); *I* is the magnitude of the electric current (A), which can be obtained by I=i⋅S, in which *i* is the current density (A/m^2^) and taken as 2.5 mA/cm^2^ in this study, and *S* is the surface area of reinforcement (m^2^).

The duration needed to reach a designed amount of mass loss can be calculated based on Faraday’s law. Therefore, the theoretical corrosion duration times of specimens with theoretical corrosion rates of *η*_t_ = 4%, *η*_t_ = 6%, and *η*_t_ = 8% were calculated to be 60 h, 90 h, and 120 h, respectively. Existing research has found that the measured corrosion levels are generally lower than the theoretical ones derived from Faraday’s law [30,31]. The comparison between the actual and theoretical corrosion levels specimens is also discussed in this paper, in Section 3.2, and the actual corrosion durations for the specimens in this study were adopted to be consistent with the theoretical ones.

**Figure 4:**
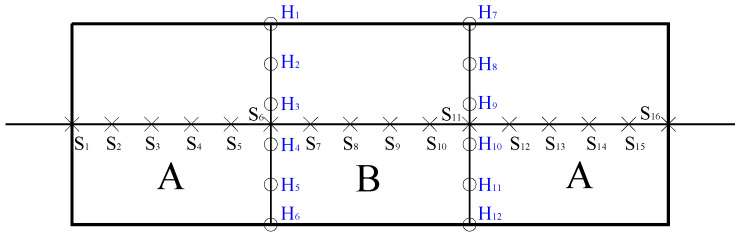
Schematic diagram of measuring points along the longitudinal and transverse cracks. (A for old concrete; B for new concrete).

The development of cracks and corrosion products accumulated on the concrete surfaces was observed during the whole accelerated-corrosion process. The crack widths on the concrete surface were measured using a ZBL-F800 crack comprehensive tester. A total of sixteen points along the longitudinal crack were selected, with a point spacing of 30 mm. The first measuring point was located at the leftmost end of the specimen where the steel bar was bonded to the concrete. Similar procedures were conducted for transverse cracks that appeared at the new-to-old concrete interface, where six points were measured with the same spacing of 30 mm, as shown in Figure 4 for segmental specimens.

### 2.3. Measurement of Corrosion Level

After finishing the accelerated corrosion test, the covering concrete was completely removed from the reinforcement and the corroded steel bars were obtained. Then, rust was cleaned from the steel bars following the treatment method provided in ASTM G1-03 [32], and then carefully weighed again by an electronic scale with a precision of 0.01 g. And the effective mass loss of the rebar $\eta_{w}$ was determined according to the measured weight before and after the corrosion process (*m*_o_ and *m*_c_, respectively), as shown in Equation (2):

(2) $$\eta_{w}=\frac{m_{o}-m_{c}}{m_{o}}\times 100\%$$

Meanwhile, the diameters of all cleaned bars were also measured with a digital display caliper with a precision of 0.01 mm. The measuring points were also selected every 30 mm along the length of the steel bar and started from the end section, where the steel bar and concrete were bonded together with a total of 16 points. These measuring points corresponded one-to-one with those along the longitudinal crack on the concrete surface. Each point on the rebar was measured twice. The two measuring positions at the same point were kept perpendicular to each other, and the average value of the two data was taken as the diameter of the point. After obtaining the diameter of the corroded steel bar, the sectional loss $\eta_{s}$ was used, as shown in Equation (3):

(3) $$\eta_{s}=\frac{S_{1}-S_{2}}{S_{1}}\times 100\%=1-{\left(\frac{d_{2}}{d_{1}}\right)}^{2}$$

where *S*_l_ is the uncorroded cross-sectional area of reinforcement (m^2^); *S*_2_ is the corroded cross-sectional area of reinforcement (m^2^); *d*_l_ is the uncorroded rebar diameter (m^2^); and *d*_2_ is the corroded rebar diameter (m^2^).

### 2.4. Tensile Tests

**Figure 5:**
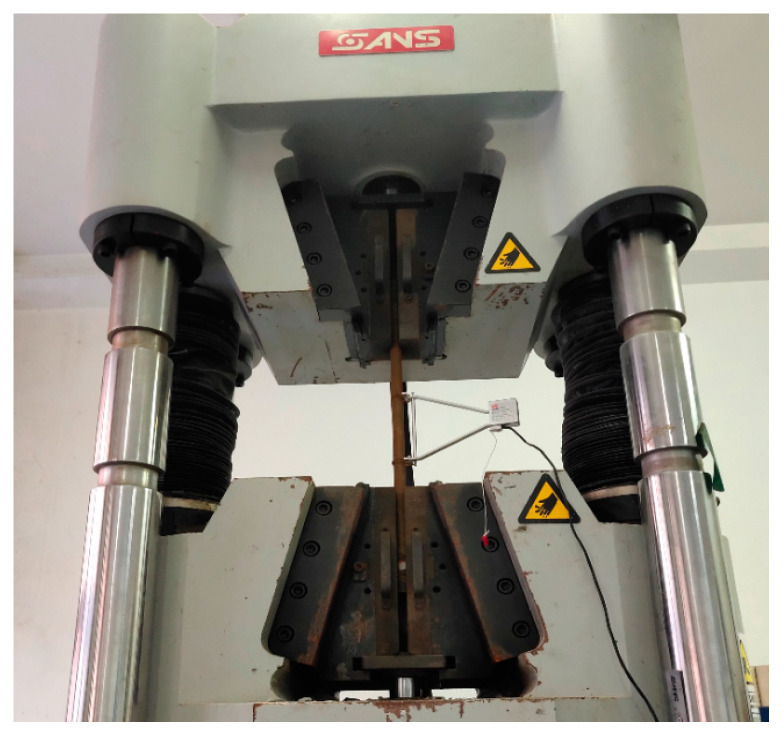
Tensile test setup.

After evaluating the corrosion levels, the corroded bars were subjected to strain-rate-controlled tensile testing according to the standard code GB/T 288.1-2021 [27] using an electro-hydraulic servo universal testing machine (see Figure 5). During the tensile test, the applied load and deformation data were collected through a computerized data acquisition system.

## 3. Test Results and Discussion

### 3.1. Corrosion Crack Development

#### 3.1.1. Monolithic Specimens

In the accelerated corrosion process, as corrosion products gradually accumulated, longitudinal cracks were observed to be generated on exposed concrete surface for the monolithic Specimens 1-b and 5-b, which were made of ordinary concrete with w/c = 0.60 and 0.53, respectively. It was observed that, when the theoretical corrosion rate of the steel bar (denoted as *η*_t_) predicted by Faraday’s Law reached 2%, local micro-cracks (with a width of about 0.08 mm) were generated on the surface of Specimen 1-b (w/c = 0.60), while there were no cracks on the surface of Specimen 5-b (w/c = 0.53).

**Figure 6:**
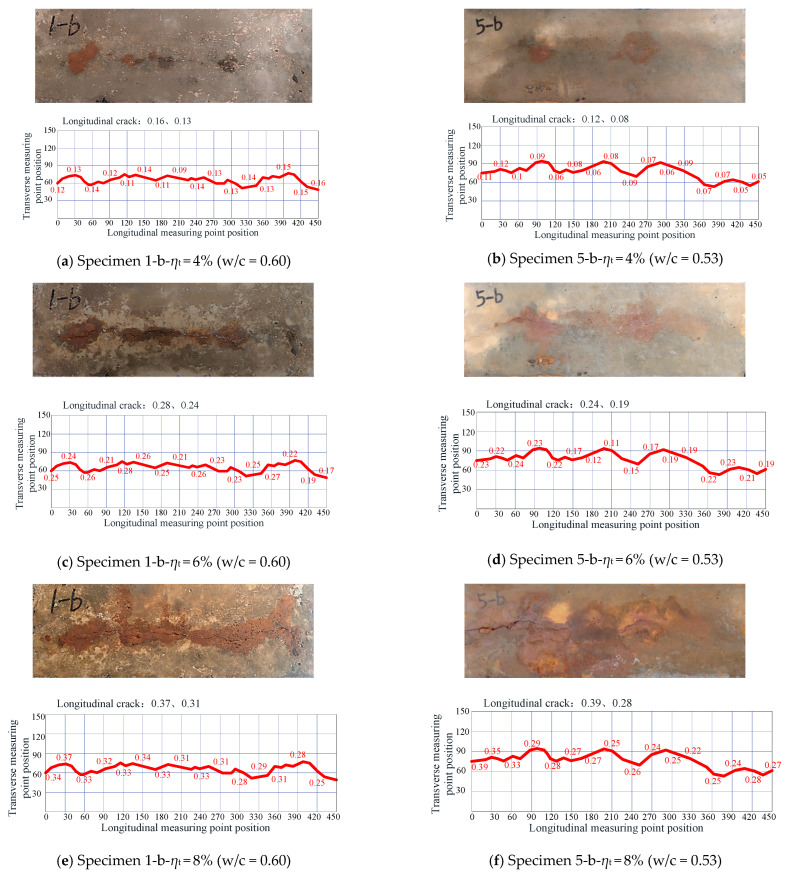
Crack development in monolithic ordinary concrete specimens (i.e., Crack widths, Unit: mm).

Figure 6 shows the crack map on the exposed concrete surface when the monolithic ordinary concrete Specimens 1-b (w/c = 0.60) and 5-b (w/c = 0.53) reached 4%, 6%, and 8% theoretical corrosion rates from Faraday’s Law, respectively. As the rust expansion cracks were very tiny, in this study, not only are the actual crack photos given in Figure 6, but the grids are also introduced to describe the crack development, and the maximum and average values of crack widths are marked in the upper-left corner of the grids. It was found that, when the theoretical corrosion rate of steel bars reached 4%, the rust expansion cracks of the ordinary concrete monolithic Specimens 1-b and 5-b penetrated the entire concrete surface, and a small number of rust products overflowed. The maximum longitudinal crack widths of Specimens 1-b and 5-b were 0.16 and 0.12 mm, respectively. When the theoretical corrosion rate reached 6%, more rust appeared on the surfaces of Specimens 1-b and 5-b, and the longitudinal cracks further developed. The maximum longitudinal crack widths of Specimens 1-b and 5-b were 0.28 and 0.24 mm, respectively. When the theoretical corrosion rate reached 8%, the corrosion products were densely spread on the entire surface, and the maximum longitudinal crack widths of Specimens 1-b and 5-b were very close to one another, with values of 0.37 and 0.39 mm, respectively. In general, the average crack widths of Specimen 1-b (w/c = 0.60 concrete) at 4%, 6%, and 8% theoretical corrosion rates were 0.13, 0.24, and 0.31 mm, respectively, which were typically larger than those of Specimen 5-b (w/c = 0.53 concrete; 0.08, 0.19, and 0.28 mm, respectively).

**Figure 7:**
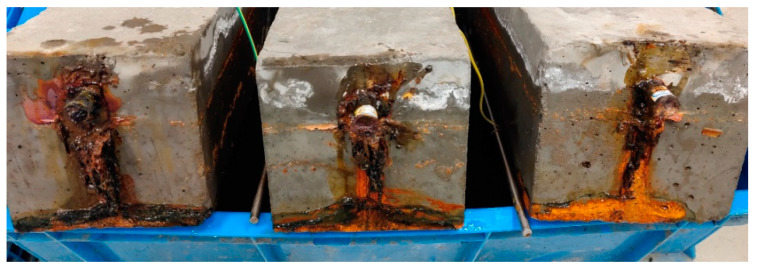
Corrosion products generated in monolithic high-strength specimens.

However, no cracks appeared on the surface of the monolithic high-strength concrete Specimens 4-a, 5-a, and 6-a when the theoretical corrosion rate reached 6%. Only corrosion products were found to leak out at the ends of the steel bars, as shown in Figure 7, with the rust being mostly black Fe_3_O_4_. This can be attributed to the fact that the permeability of high-strength concrete was greatly improved by the use of a lower water–cement ratio; thus, the oxygen supply required in the steel corrosion process was not sufficient, and the volume expansion coefficient of the generated corrosion products was less than that in ordinary concrete. This means that the rust expansion force generated by the corrosion products was not large enough to crack the concrete cover compared with the tensile strength of high-strength concrete. Therefore, the corrosion products could only leak out from the longitudinal channels along the rebar.

#### 3.1.2. Segmental Specimens with New-to-Old Concrete Interface

**Figure 8:**
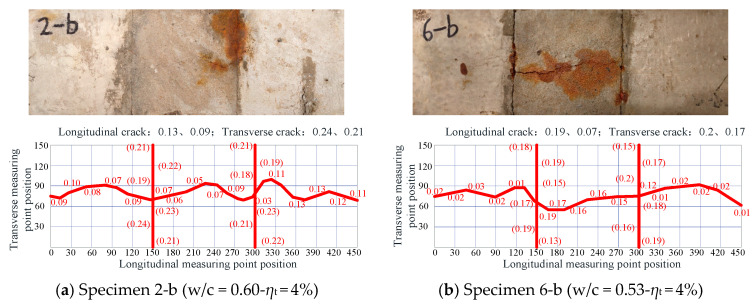

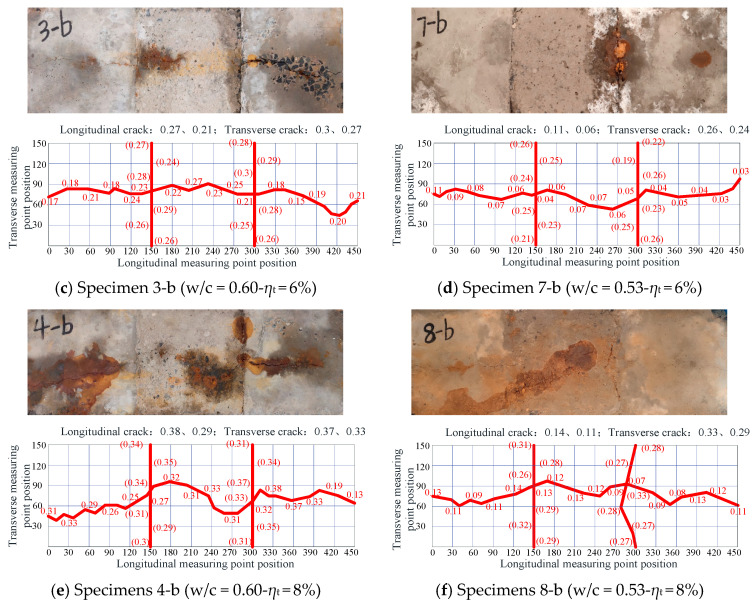
Crack development in segmental ordinary concrete specimens (Unit: mm).

For segmental specimens produced with ordinary concrete, transverse cracks first appeared on the new-to-old concrete interface, followed by longitudinal cracks on the concrete surface along the rebar. Moreover, longitudinal cracks always appeared first on the new concrete surface (Part B in Figure 1), which were usually wider than those on the old concrete’s surface. The cracks generated on the segmental ordinary concrete specimens are shown in Figure 8. The results indicated that, when the steel embedded in concrete reached the theoretical corrosion rates of 4%, 6%, and 8%, both the average longitudinal and transverse cracks in the segmental Specimens 2-b, 3-b, and 4-b (w/c = 0.60) were wider than those in Specimens 6-b, 7-b, and 8-b (w/c = 0.53). The longitudinal cracks developed faster than transverse cracks. The comparison between the monolithic and segmental specimens with ordinary concrete indicated that the average longitudinal cracks on the surface of the segmental specimens were much narrower than those of monolithic specimens, mainly due to expansion stress being released through transverse cracks in the segmental specimens.

**Figure 9:**
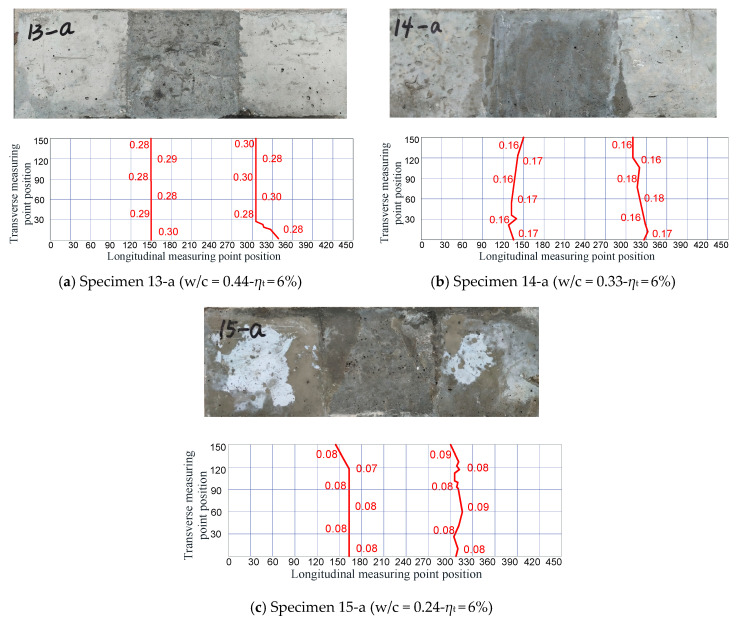
Crack development in segmental high-strength concrete specimens (Unit: mm).

For the segmental high-strength concrete specimens, it was observed that, at the beginning of electrification, a small amount of rust leaked out from two ends. As corrosion continued, there was no longer any leakage of corrosion products from both ends of the specimen. Cracks first appeared on the middle part of new-to-old concrete interfaces, then propagated to both sides of the interface, and finally penetrated through the entire interface, resulting in a transverse crack. The crack development in segmental high-strength concrete specimens under a specified corrosion level of *η*_t_ = 6% is shown in Figure 9. It can be seen that the corrosion products generated on the concrete surface for the segmental specimens made with ordinary concrete and high-strength concrete were different. The corrosion products of steels in ordinary concrete were mostly reddish-brown ferric hydroxide, but those in high-strength concrete were mostly black ferro ferric oxide. This is consistent with the phenomenon presented in the monolithic specimens.

**Figure 10:**
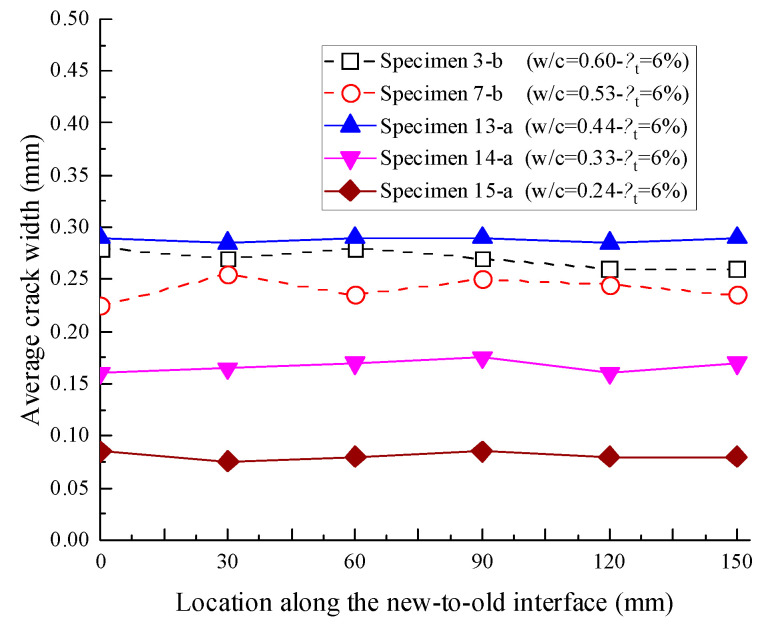
Average widths of transverse cracks at each location in the segmental specimens.

As shown in Figure 10. for Specimens 13-a, 14-a, and 15-a under the same corrosion level *η*_t_ = 6%, the results indicated that the average transverse crack widths at the new-to-old interface tended to decrease with decreasing water–cement ratios; here, the average width of transverse cracks was denoted as the average value of two transverse cracks at the same location. The only exception was Specimen 13-a, with a water–cement ratio of 0.44, whose average transverse crack widths were larger than those of Specimens 3-b and 7-b, even though their water–cement ratios were both larger than that of Specimen 13-a. The reason is that, although fewer corrosion products were accumulated in Specimen 13-a compared with those in Specimens 3-b and 7-b, only transverse cracks were generated on Specimen 13-a. Thus, the rust in Specimen 13-a could only overflow from the new-to-old concrete interfaces.

**Figure 11:**
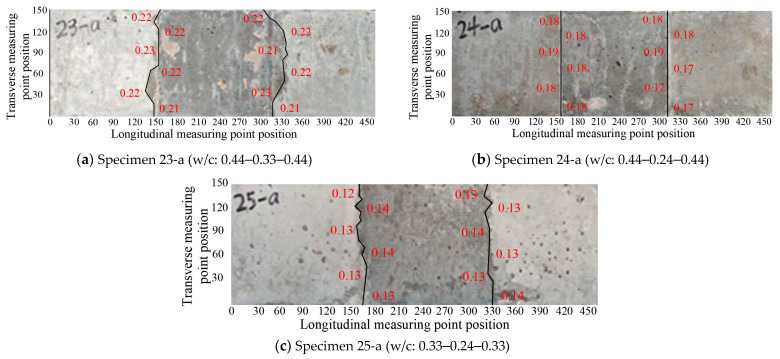
Crack development of segmental specimens with the strength difference between new and old concrete.

**Figure 12:**
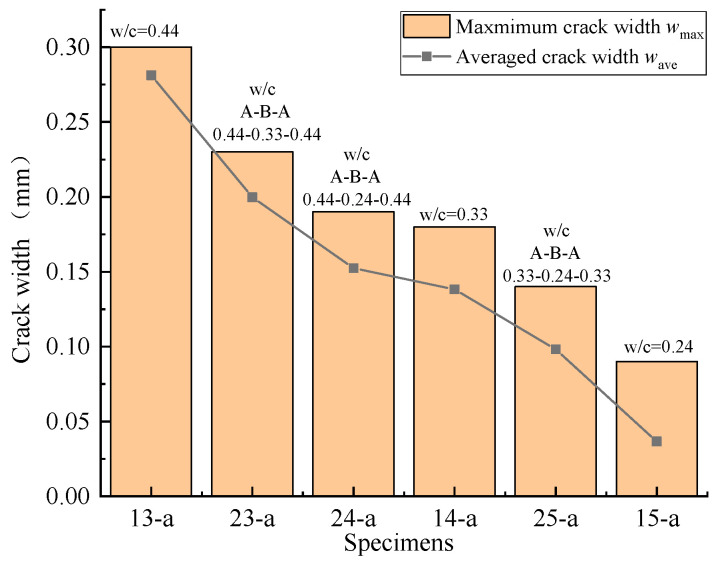
Influence of the strength difference between new and old concrete on transverse crack widths.

As mentioned before, Specimens 23-a, 24-a, and 25-a were designed to take into account the difference in strength between new and old concrete. The development of concrete surface cracking is shown in Figure 11. Just like the other segmental specimens with high-strength concrete, only transverse cracks appeared on the concrete surface, which basically coincided with the interfaces between the new and old concrete. When comparing Specimen 23-a with Specimens 13-a and 14-a, Specimen 14-a had the highest strength, followed by Specimen 23-a, and Specimen 13-a had the lowest strength. The corresponding maximum crack widths for Specimens 23-a, 13-a, and 14-a were observed to be 0.23, 0.30, and 0.18 mm, respectively, and their average crack widths were 0.219, 0.288, and 0.167 mm, respectively, which conformed to the same trend; that is, the lower the concrete strength, the larger the crack width. Similar trends were observed for Specimen 24-a compared with Specimens 13-a and 15-a, and for Specimen 25-a compared with Specimens 14-a and 15-a (Figure 12).

### 3.2. Corrosion Level of Reinforcement

#### 3.2.1. Sectional Loss of Reinforcement

**Figure 13:**
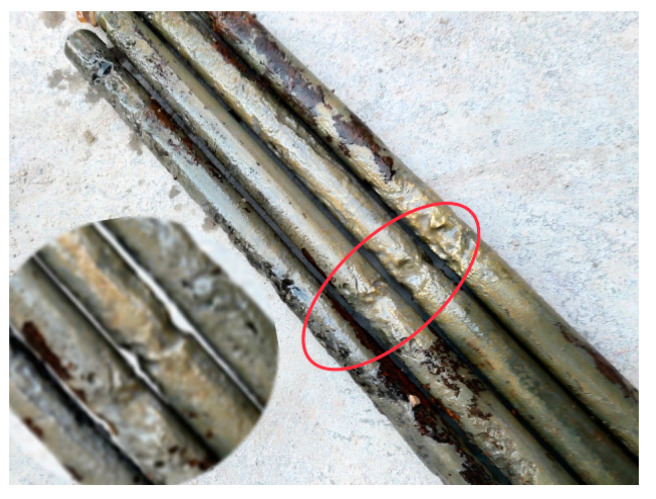
Reinforcement cross-section losses.

Figure 13 shows a photo of the pickled corroded steel bars, with the leftmost bar embedded in the monolithic specimen and the others embedded in the segmental specimens. A uniform distribution of cross-sectional areas of corroded bars was observed in monolithic specimens. In contrast, the corroded bars in segmental specimens showed non-uniform corrosion, with maximum sectional loss occurring at the new–old concrete interface due to the cracks generated there providing a channel for the transfer of oxygen and moisture from outside to the steel surface. It resulted in more iron losing electrons to participate in the electrochemical reaction at the new-to-old concrete interface, causing a larger loss of its cross-section at the interface.

**Figure 14:**
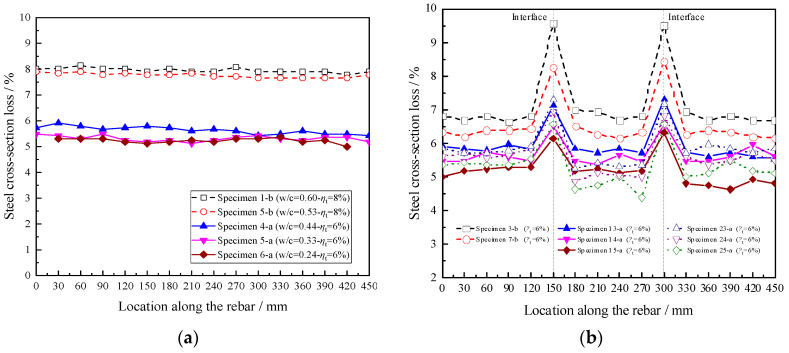
Cross-section losses of corroded steel: (**a**) Monolithic specimens; (**b**) Segmental specimens.

Figure 14 shows the cross-section losses along the corroded rebar for specimens. It was found that, for monolithic specimens (i.e., Specimens 1-b, 5-b, 4-a, 5-a, and 6-a), the cross-section losses were evenly distributed along the entire length of the reinforcement, as shown in Figure 14a. The average sectional losses for Specimens 1-b and 5-b, with ordinary concrete under 8% theoretical corrosion rate, were 7.96% and 7.76%, respectively, while the corresponding values for Specimens 4-a, 5-a, and 6-a, with high-strength concrete under 6% theoretical corrosion rate, were 5.63%, 5.31%, and 5.23%, respectively. The results indicate that the average cross-section losses in the monolithic specimens are smaller than the theoretical corrosion rate, but these two values were very close and their largest difference was no more than 13%. Additionally, the difference between these two values increased with the decrease in the water–cement ratio.

For the segmental specimens, the most serious loss in the section of the steel occurred at the new-to-old concrete interfaces. This is consistent with the study conducted by Liu et al. [18]. The maximum sectional losses of steel bars for Specimens 3-b, 7-b, 13-a, 14-a, and 15-a under a 6% theoretical corrosion rate were 9.57%, 8.44%, 7.30%, 6.58%, and 6.33%, respectively, as given in Figure 14b. Among them, Specimen 3-b (w/c = 0.60) suffered the largest sectional loss of steel, and its maximum, minimum, and average reinforcement sectional losses were 9.57%, 6.64%, and 7.14%, respectively, with the difference between the maximum and minimum sectional losses reaching up to 44.2%. The change in the water–cement ratio had a significant influence on the reinforcement cross-section loss for the segmental specimens. It decreased sharply with the decrease in the water–cement ratio.

In addition, for Specimens 23-a, 24-a, and 25-a, with the strength of the new concrete (i.e., Part B) being higher than that of the old concrete (i.e., Part A), it was observed that, in the segments outside the interfaces, the cross-sectional loss of steel bars in part A was generally higher than that in part B. Compared with Specimens 24-a and 23-a, Specimen 25-a exhibited the lowest values in both average and maximum cross-sectional losses.

Huang et al. [25] found that the corrosion of steel bars at the joint sections was significantly higher than that of non-joint sections. The corrosion rate of steel bars may not effectively reflect this uneven phenomenon. Therefore, they proposed an influence coefficient (*k*_j_) of construction joints on the local weight loss to reflect the influence of joints on the rust uniformity of rebars, which was defined as follows:

(4) $$k_{j}=\frac{2m_{2}}{m_{1}+m_{3}}$$

where *m*_2_ is the rust amount of the rebar in joint section (g); *m*_1_ is the rust amount of the rebar in the non-joint new concrete section (g); and *m*_3_ is the rust amount of the rebar in non-joint old concrete section (g). The values of *k*_j_ were in the range of 1.15–3.33, with an average of 1.76.

Considering that the cross-sectional loss measurement of steel bars in this study was more detailed than mass loss measurement, an influence coefficient *k*_s_ based on the section loss of steel bars was proposed to evaluate the influence of joints on the rust uniformity of rebars, as defined below:

(5) $$k_{s}=\frac{S_{i1}+S_{i2}}{S_{n}+S_{o}}$$

where *S*_i_ is the section loss of the rebar at the new-to-old concrete interface; in this study, there were two interfaces; thus, it was the sum of the two, i.e., *S*_i_ = *S*_i1_ + *S*_i2_; *S*_n_ and *S*_o_ were the average section losses of the rebar at each measuring point in the new and old concrete sections, respectively.

The influence coefficients *k*_s_ of all segmental specimens under the theoretical corrosion rate of 6% are listed in Table 3. The values of *k*_s_ were in the range of 1.180–1.401, with an average value of 1.286. The maximum value of *k*_s_ was 1.401 for Specimen 3-b and the minimum was 1.180 for Specimen 14-a. The results show that, when the new and old concrete sections had the same mix proportion, the values of *k*_s_ decreased almost linearly with the decrease in the water–cement ratio, which means that increasing the concrete strength could reduce the non-uniformity of steel corrosion due to the presence of the joint. But when there were differences in strength between the new and old concrete, the influence of the interface on the uniformity of steel bar cross-section loss slightly increased. For example, compared with Specimens 13-a and 14-a, the *k*_s_ value of Specimen 23-a increased by 4.00% and 10.08%, respectively. A similar trend was found in Specimens 25-a and 24-a.

#### 3.2.2. Comparison between Actual and Theoretical Corrosion Levels of Steel

In this section, more attention was paid to three parameters obtained in the experiments to measure the corrosion level of steel, namely the mass loss (*η*_w_), the average cross-section loss (*η*_s-ave_), and maximum cross-section loss (*η*_s-max_) of the corroded steel. The relationship between these three parameters and the theoretical corrosion rate (*η*_t_) predicted from Faraday’s Law was analyzed in detail.

**Figure 15:**
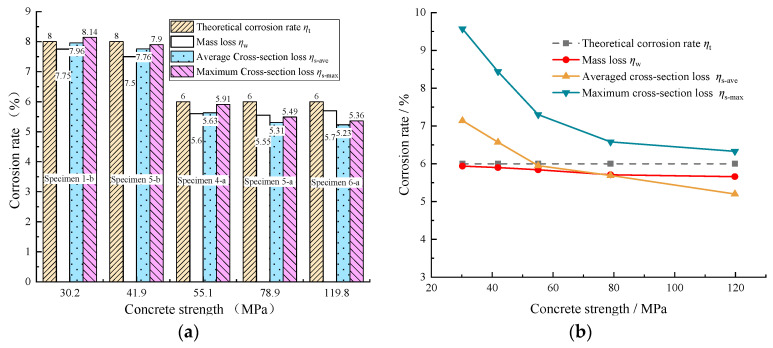
Comparison of corrosion rates of reinforcement: (**a**) Monolithic specimens; (**b**) Segmental specimens.

Figure 15a,b show the comparison of these three parameters with the theoretical corrosion rates for the monolithic Specimens 1-b, 5-b, 4-a, 5-a, and 6-a and the segmental Specimens 3-b, 7-b, 13-a, 14-a, and 15-a, respectively. The results in Figure 15a show that these three parameters in the monolithic specimens were relatively close to the theoretical corrosion rate, with the maximum difference being no more than 13%. Moreover, both the mass and average cross-section losses of the steel in monolithic specimens were lower than the theoretical corrosion rate. But in the segmental specimens, as shown in Figure 15b, only the mass loss of the steel was a bit lower than the theoretical corrosion rate, with a maximum difference of no more than 6%. Since the theoretical corrosion rate derived from Faraday’s Law represents the mass loss of steel, by comparing the values of the mass loss of steel from the experimental study and Faraday’s Law, it can be acknowledged that the findings in this study are consistent with those of previous studies [30,31], which showed that the measured mass loss was generally lower than the theoretical one derived from Faraday’s Law. On the other hand, the average and maximum cross-sectional loss of the steel in the segmental specimens varied greatly, with maximum differences of up to 19.0% and 59.5%, respectively, concerning the theoretical corrosion rate of the steel (*η*_t_ = 6%). It is also important to note that the maximum sectional loss of steel for each segmental specimen was larger than the theoretical corrosion rate predicted from Faraday’s Law (*η*_t_ = 6%). The ratios between the measured maximum cross-section losses of steel to the theoretical corrosion rates from Faraday’s Law ranged from 1.05 to 1.6. Thus, for the segmental specimens, sectional loss of steel is suggested to represent the corrosion level of steel.

**Figure 16:**
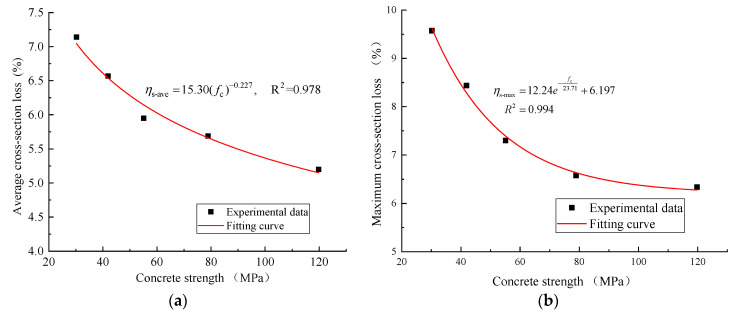
Variation in the average and maximum sectional losses of steel with concrete strength in segmental specimens: (**a**) Average cross-section loss; (**b**) Maximum cross-section loss.

Figure 16a,b show the variation in the average and maximum cross-section loss of the steel with the concrete strength in the segmental specimens. The results demonstrated that a power relationship linking the average cross-sectional loss of the steel $\eta_{s-\mathrm{ave}}$(%) and concrete strength $f_{c}$(MPa) could be proposed for the segmental specimens, as follows:

(6) $$\eta_{s-\mathrm{ave}}=15.30{\left(f_{c}\right)}^{-0.227},R^{2}=0.978$$

And an exponential relationship could be established between the maximum reinforcement cross-sectional loss $\eta_{s-\max}$(%) and concrete strength $f_{c}$(MPa) for the segmental specimens with the following Equation (7):

(7) $$\eta_{s-\max}=12.24e^{-\frac{f_{c}}{23.71}}+6.197\;\;R^{2}=0.994$$

#### 3.2.3. Mechanical Properties of Corroded Reinforcement

**Figure 17:**
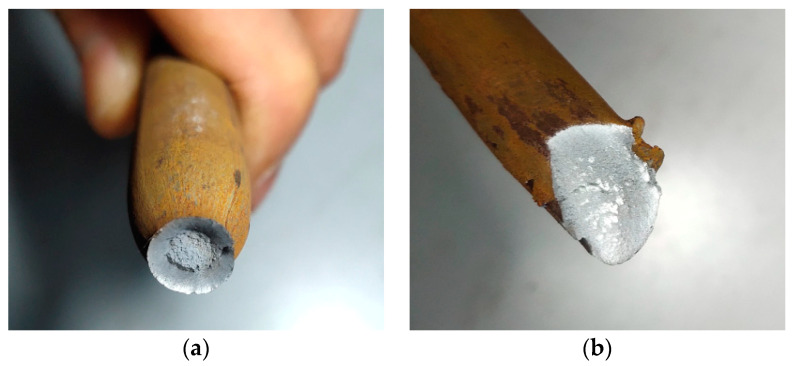
Fracture type of corroded reinforcement: (**a**) monolithic specimens; (**b**) segmental specimens.

Tensile tests were performed on corroded reinforcements. The results indicated that rebars in monolithic specimens fractured at the middle section due to uniform cross-section losses, while rebars in segmental specimens fractured at the minimum cross-sections, i.e., at the new-to-old concrete interfaces. Figure 17a,b show the tensile failure of the reinforcements in the monolithic and segmental specimens, respectively. The reinforcements in monolithic specimens mostly necked before failure, whereas those in the segmental specimens showed brittle fracture due to non-uniform corrosion, with the maximum cross-section loss at the new-to-old concrete interface.

Until now, different methods have been used to assess the strength properties, such as nominal (apparent) strength and residual (or true) strength [33]; that is, the tensile strength of corroded bars can be calculated from the measured force based on the nominal steel area, the average residual steel area, and the residual minimum steel area [34,35,36]. Since no consensus has been reached among researchers regarding which method is more appropriate than the others [37], in this study, the yield and ultimate strengths of corroded bars were calculated with the nominal cross-sectional area of the original steel bar. The corresponding tensile behaviors of the nominal yield strength, *f*_yc_, and nominal ultimate strength, *f*_uc_, of corroded steel bars are listed in Table 4.

Corroded steels in segmental specimens showed a larger decrease in nominal ultimate strength than those in monolithic specimens. Concrete strength significantly affected the nominal ultimate strength of corroded steels in both monolithic and segmental specimens. The reduction in ultimate strength decreased with rising concrete strength, but the trend was less evident for yield strength, which could be influenced by various factors besides the minimum cross-sectional area.

It is also worth mentioning that, when using ordinary concrete with a water–cement ratio of 0.60 and 0.53 for all specimens under the theoretical corrosion rate of 6%, both the yield and ultimate stresses of corroded steels decreased greatly by 12.10–16.56%. However, when high-performance concrete with water–cement ratios of 0.44, 0.33, and 0.24 was used for all specimens under the theoretical corrosion rate of 6%, both the yield and ultimate stresses of the rusted steel only slightly decreased by 2.37–7.01%. This implies that the use of high-strength concrete can effectively reduce the strength loss of corroded steels, because the water-cement ratio affects the permeability of concrete. For segmental specimens, the tensile behavior of corroded steels largely depended on the steel corrosion at the new-to-old concrete interface, and the concrete type was an important factor affecting the bonding performance at the new-to-old concrete interface.

**Figure 18:**
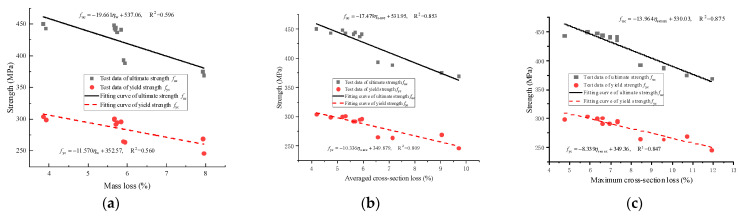
Variation in the nominal yield and ultimate strength with corrosion level of steel in segmental specimens: (**a**) Mass loss; (**b**) Average cross-section loss; (**c**) Maximum cross-section loss.

Furthermore, for the segmental specimens, it was found that the correlation between the maximum sectional loss and the tensile behavior of corroded steel was the strongest, as shown in Figure 18a–c. A linear function could be established between the maximum sectional loss and the nominal yield strength *f*_yc_ (or the nominal ultimate strength *f*_uc_) of the corroded steel. It was more appropriate to use cross-section loss to evaluate steel corrosion in segmental specimens with the new-to-old concrete interfaces.

### 3.3. Prediction of Concrete Cracking Time

This paper refers to the expression proposed by Jin et al. [38] for uniform corrosion-induced stress *q* in smooth steel bars when concrete cracking was initiated based on the theory of elastic mechanics:

(8) $$\;q=\left(0.3+0.6\frac{c}{d}\right)f_{\mathrm{tk}}$$

where *c* is the net concrete cover thickness (in this study *c* = 25 mm), *d* is the rebar diameter (*d* = 16 mm), and *f*_tk_ is the standard value of axial tensile strength. Thus, the uniform corrosion-induced stresses when concrete cracking was initiated for the monolithic Specimen 1-b (*f*_tk_ = 2.01 MPa) with w/c = 0.60 and Specimen 5-b (*f*_tk_ =2.40 MPa) with w/c = 0.53 were 2.49 MPa and 2.97 MPa, respectively.

Jin et al. [38] also proposed a simplified formula to express the uniform corrosion-induced stress *q* in rebars prior to concrete expansion cracking, which was expressed as a function of the steel corrosion rate *ρ*:

(9) $$\;q=35\delta =35\left(\sqrt{\left(n-1\right)\rho +1}-1\right)R$$

where *δ* is the thickness of the rust layer; *n* is the volume expansion ratio of the corroded rebar, typically ranging between 2 and 4; *ρ* is the corrosion rate of the rebar, evaluated using the cross-sectional loss percentages; and *R* is the rebar radius.

The experimental results in Figure 15a demonstrate that the theoretical corrosion rate of steel reinforcement for the monolithic specimens exhibited little difference from the cross-sectional loss (as discussed in Section 3.2.2). Moreover, for the monolithic specimens, black–brown rust products were observed to overflow from both sides of the steel bars; thus, the main rust product was Fe_3_O_4_. Referring to the volume expansion ratio of the corrosion products given by Ji et al. [39], *n* was set to be 2.0.

Combining Equations (8) and (9), the predicted cross-sectional losses for the monolithic Specimens 1-b and 5-b when concrete cracking was initiated were 1.78% and 2.13%, respectively, consistent with the experimental observation in Section 3.1.1; that is, when the steel theoretical corrosion rate reached 2%, local micro-cracking was generated on the monolithic Specimen 1-b (w/c = 0.60), while no cracking was observed on the concrete surface of the monolithic Specimen 5-b (w/c = 0.53).

According to Faraday’s law in Equation (1), the time for concrete cracking initiation can be predicted as follows:

(10) $$t=\frac{\Delta m_{L}\;\cdot \;Z\;\cdot \;F}{M\;\cdot \;I}$$

By substituting the cross-sectional losses obtained for the monolithic Specimens 1-b and 5-b when concrete cracking initiated into Equation (10), the predicted concrete cracking times for the monolithic Specimens 1-b and 5-b were 26.8 h and 32.0 h, respectively.

Through theoretical calculation, it was found that the cross-sectional loss predicted for concrete cracking initiation in monolithic specimens was basically consistent with experimental phenomena. However, because the focus of this study was on concrete surface cracking and the subsequent mechanical properties of corroded rebars, the initial cracking time was not monitored, so a comparison between the predicted initial cracking time and the actual one was not possible.

As for the segmental specimens, non-uniform corrosion-induced rust expansion should be considered. It is necessary to combine experiments and numerical simulation to obtain relevant data, such as the time for concrete cracking, the corresponding cross-sectional loss of steel bars, and the non-uniform rust expansion pressure at concrete cracking. It is also possible to combine existing theoretical non-uniform corrosion models for analysis, and thus to achieve the prediction of concrete cracking time for segmental specimens. This needs further research in the future.

## 4. Conclusions

Monolithic and segmental specimens with new-to-old concrete interfaces were tested to investigate their concrete surface cracking based on the impressed current-accelerated corrosion method. Moreover, the steel corrosion and mechanical properties of the corroded steels were observed and examined for their differences in the two kinds of specimens. Important conclusions are summarized as follows:

In the accelerated corrosion process, for the monolithic specimens, longitudinal cracks were generated on the ordinary concrete surface, whereas no cracks were produced on the high-strength concrete surface; only the rust products leaked out at the ends. For the segmental specimens, both longitudinal and transverse cracks were generated on the ordinary concrete surface, whereas only transverse cracks appeared on the high-strength new-to-old concrete interfaces.

(1) The steels embedded in segmental specimens suffered higher cross-sectional loss at the new-to-old concrete interfaces, with the maximum sectional loss of the steel sharply decreasing as the concrete strength increased. An influence coefficient based on the section loss of the rebar was proposed to evaluate the influence of interfaces on the rust uniformity of rebars. When there were differences in strength between new and old concrete, the influence of the interface on the uniformity of the steel bar cross-section loss slightly increased.
(2) Both the mass loss and sectional loss could be used to describe the steel uniform corrosion in the monolithic specimens, but for the segmental specimens, it was more appropriate to use cross-sectional loss to evaluate steel corrosion, due to the steel at the new-to-old concrete interface suffering worse sectional loss.
(3) The corroded steels embedded in the monolithic specimens fractured at the middle section, while those embedded in the segmental specimens fractured at the new-to-old concrete interface. The corroded steel bars in the segmental specimens showed worse tensile behavior compared with those in the monolithic specimens.
(4) Based on available theoretical analysis for uniform corrosion, the concrete cracking time of the monolithic specimens was predicted, which was basically consistent with experimental phenomena. However, further research is needed to predict the service life of segmental specimens.

Since accelerated corrosion only lasts for a few tens of hours, corrosion products do not have enough time to invade the concrete pores. In future studies, wet–dry cycling tests can be carried out to simulate the real environment to study the cracking behavior of the concrete protective layer in new and old concrete structures. The cracking of new-to-old concrete under the coupling of carbonation and other factors can also be studied in depth to better understand the reaction kinetics, diffusion mechanisms, and influencing factors. Secondly, this experiment only considered the effects of the pouring method, concrete strength, and strength difference between new and old concrete. Other design parameters such as the concrete cover thickness, the type and quantity of steel bars, and the interface treatment, can be considered in the future to reveal the rust cracking process caused by the presence of new-to-old concrete interfaces. Furthermore, some microscopic detection methods, such as slicing and optical scanning, should be introduced to study the rust expansion behavior and achieve service life prediction for RC structures with new-to-old concrete interfaces.

## Figures and Tables

**Table 1:** Features of the specimens.

Group	Specimens	Casting Method	Theoretical Corrosion Rate *η*_t_ (%)	Water–Cement Ratio		Concrete Number
				Monolithic	Segmental Casting (A–B–A)	
Ordinary concrete	1-b	Monolithic	8	0.6		No.1
	2-b	Segmental casting	4	/	0.60–0.60–0.60	No.1
	3-b	Segmental casting	6		0.60–0.60–0.60	No.1
	4-b	Segmental casting	8		0.60–0.60–0.60	No.1
	5-b	Monolithic	8	0.53		No.2
	6-b	Segmental casting	4	/	0.53–0.53–0.53	No.2
	7-b	Segmental casting	6		0.53–0.53–0.53	No.2
	8-b	Segmental casting	8		0.53–0.53–0.53	No.2
High-strength concrete	4-a	Monolithic	6	0.44		No.3
	5-a	Monolithic	6	0.33		No.4
	6-a	Monolithic	6	0.24		No.5
	13-a	Segmental casting	6	/	0.44–0.44–0.44	No.3
	14-a	Segmental casting	6		0.33–0.33–0.33	No.4
	15-a	Segmental casting	6		0.24–0.24–0.24	No.5
	23-a	Segmental casting	6		0.44–0.33–0.44	No.3 and No.4
	24-a	Segmental casting	6		0.44–0.24–0.44	No.3 and No.5
	25-a	Segmental casting	6		0.33–0.24–0.33	No.4 and No.5

**Table 2:** Mix proportions and properties of concrete.

Number	1	2	3	4	5
Cement type	P.O42.5	P.O42.5	P.O42.5	P.II52.5	P.II52.5
Cement quantity (kg/m^3^)	350	300	350	440	550
Slag type	/	S95	S95	S95	S95
Slag quantity (kg/m^3^)	/	60	70	60	60
Fly ash type	/	F-I	F-I	F-I	F-I
Fly ash quantity (kg/m^3^)	/	60	70	60	70
Fine aggregate (medium sand) quantity (kg/m^3^)	719	895	867	855	795
Coarse aggregate (size: 5–10 mm) quantity (kg/m^3^)	1170	564	542	513	477
Coarse aggregate (size: 10–20 mm) quantity (kg/m^3^)		376	361	342	318
Admixture Type	/	DSZ-1	DSZ-1	DSZ-1	DSZ-1
Admixture quantity (kg/m^3^)	/	2.80	3.92	10.08	18.9
Water quantity (kg/m^3^)	210	160	155	145	130
w/c ratio	0.60	0.53	0.44	0.33	0.24
Compressive strength (MPa)	30.2	41.9	55.1	78.9	119.8

**Table 3:** The influence coefficient *k*_s_ of the interface.

Specimens	3-b	7-b	13-a	14-a	15-a	25-a	24-a	23-a
w/c	0.60	0.53	0.44	0.33	0.24	0.44–0.33–0.44	0.44–0.24–0.44	0.33–0.24–0.33
*k*_s_	1.401	1.322	1.249	1.180	1.227	1.316	1.295	1.299

**Table 4:** Properties of corroded steel bars.

Specimens	Water–Cement Ratio	Casting Method	Theoretical Corrosion Rate *η*/%	Mass Loss Ratio *η*_w_/%	Averaged Sectional Loss Ratio *η*_s-ave_/%	Maximum Sectional Loss Ratio *η*_s-max_/%	Yield Strength *f*_yc_/MPa	Ultimate Strength *f*_uc_/MPa
1-b	0.6	Monolithic	8	7.75	7.96	8.14	274	394
5-b	0.53	Monolithic	8	7.5	7.76	7.9	276	395
4-a	0.44	Monolithic	6	5.6	5.63	5.91	305	449
5-a	0.33	Monolithic	6	5.55	5.31	5.49	305	452
6-a	0.24	Monolithic	6	5.7	5.23	5.36	294	454
2-b	0.6–0.6–0.6	Segmental	4	3.92	4.74	6.82	299	443
3-b	0.6–0.6–0.6	Segmental	6	5.94	7.14	9.57	264	388
4-b	0.6–0.6–0.6	Segmental	8	7.96	9.71	11.93	246	369
6-b	0.53–0.53–0.53	Segmental	4	3.85	4.19	5.85	304	450
7-b	0.53–0.53–0.53	Segmental	6	5.9	6.57	8.44	265	393
8-b	0.53–0.53–0.53	Segmental	8	7.93	9.04	10.7	269	375
13-a	0.44–0.44–0.44	Segmental	6	5.84	5.95	7.3	296	441
14-a	0.33–0.33–0.33	Segmental	6	5.71	5.69	6.58	292	444
15-a	0.24–0.24–0.24	Segmental	6	5.66	5.20	6.33	300	448
23-a	0.44–0.33–0.44	Segmental	6	5.74	5.87	7.30	295	437
24-a	0.44–0.24–0.44	Segmental	6	5.70	5.62	6.94	292	441
25-a	0.33–0.24–0.33	Segmental	6	5.67	5.31	6.58	301	443

Note: for the uncorroded steel: *f*_y0_ = 314 MPa, *f*_u0_ = 465 MPa.

## Data Availability

Data sharing not applicable.

## Abbreviation

*f*_y_	characteristic steel yield strength	*k*_j_	influence coefficient of construction joints
Δ*m*_L_	mass loss of iron (g)	*m*_2_	rust amount of the rebar in joint section (g)
*M*	molar mass of iron (56 g/mol for Fe)	*m*_1_	rust amount of the rebar in non-joint new concrete section (g)
*Z*	valency of the iron element (i.e., 2.5)	*m*_3_	rust amount of the rebar in non-joint old concrete section (g)
*F*	Faraday constant (i.e., 96,500 C/mol)	*k*_s_	influence coefficient based on section loss of steel bars
*t*	duration of electrochemical corrosion(s)	*S*_i_	section loss of the rebar at the new-to-old concrete interface
*I*	magnitude of the electric current (A)	*S*_n_ *, S*_o_	average section loss of the rebar at each measuring point in the new and old concrete sections, respectively.
*S*	surface area of reinforcement (m^2^)	*η*_w_	mass loss of the corroded steel
*η*_t_	theoretical corrosion rate	*η*_s-ave_	average cross-section loss of the corroded steel
$\eta_{w}$	effective mass loss of the rebar	*η*_s-max_	maximum cross-section loss of the corroded steel
*m*_o_	measured weight before the corrosion process	*q*	uniform corrosion-induced stress
*m*_c_	measured weight after the corrosion process	*c*	net concrete cover thickness
$\eta_{s}$	sectional loss	*d*	rebar diameter
*S*_l_	uncorroded cross-sectional area of reinforcement (m^2^)	*f*_tk_	standard value of axial tensile strength.
*S*_2_	corroded cross-sectional area of reinforcement (m^2^)	δ	thickness of the rust layer
*d*_l_	uncorroded rebar diameter (m^2^)	n	volume expansion ratio of the corroded rebar
*d*_2_	corroded rebar diameter (m^2^)	*ρ*	corrosion rate of the rebar, evaluated using the cross-sectional loss percentages
*η*_s-max_	maximum cross-section loss of the corroded steel		
